# Characterization of Bioactive Compounds in Lees from New Zealand Wines with Different Vinification Backgrounds

**DOI:** 10.3390/antiox11122335

**Published:** 2022-11-25

**Authors:** Zhijing Ye, Yunxuan Qin, Roland Harrison, Richard Hider, Alaa El-Din A. Bekhit

**Affiliations:** 1 School of Viticulture and Wine, Eastern Institute of Technology, Te Pūkenga, Trading as EIT, Napier 4112, New Zealand; 2 Department of Wine, Food and Molecular Biosciences, Lincoln University, Lincoln 7647, New Zealand; 3 Department of Food Science, The University of Otago, Dunedin 9054, New Zealand

**Keywords:** wine lees, phenolic compounds, antioxidant, antimicrobial activity, vinification

## Abstract

Wine lees are one of the main by-products produced during winemaking. Little is known about the effect of the vinification technique on the phenolic compounds and the biological activity of wine lees extracts. Wine lees collected at varying vinification sources of two grape varieties, Riesling (RL) and Pinot Noir (PN), were analyzed for total phenolic content (TPC), tannin content (TTC), their anthocyanin and phenolic profile, and the antioxidant and antimicrobial activities of their extracts. The results showed a low TPC and TTC in RL lees, which could be attributed to the varietal characteristic of RL grapes and to less skin contact during vinification. Vinification techniques modified the composition of the phenolic compounds in the lees. The results showed a good linear relationship between the antioxidant activities and the TPC and TTC, indicating that PN lees were better sources of phenolics and antioxidant activity than RL lees. The antimicrobial activity of wine lees was related to the phenolic composition rather than the quantity of total phenolics. Knowing the grape and wine processing conditions can provide some insights into the potential composition of wine lees and, hence, determine the potential economic use of the by-product.

## 1. Introduction

Significant growth in the New Zealand wine industry started about three decades ago. As of June 2019, the New Zealand wine industry recorded its 24th consecutive year of export growth [1]. While enjoying its great success as a well-established member of the “New World” wine producing country, the expansion of the industry has experienced several negative issues in terms of the by-products generated from the industry and consequent waste management issues.

According to Lavelli et al. [2], 100 kg of processed grapes generates approximately 20–25 kg of pomace (also known as grape marc), 3–5 kg of stalks, and 8–10 kg of lees, depending on the grape variety and the vinification methods applied. It is important to develop new strategies to reduce the waste, or by-products, generated from winemaking by using an added value approach since waste minimization is crucial to maintain the industry’s clean and green image. In recent years, great interests and efforts have been made to characterize and manage the grape marc (i.e., a mixture of grape skins and seeds) with the aim to use their extracts as health supplements. For example, Shrikhande [3] and González-Paramás et al. [4] reported that extracts from grape seeds and skin contain phenolic compounds and can be used as dietary supplements for better health and well-being. However, the investigation of wine lees generated in post-fermentation is poorly studied with very little information available in the literature despite the fact that lees contribute to about 14% of the total organic waste produced from wine production [5].

Wine lees are sludge (mud-like) materials generated in various processes, such as after fermentation, during storage or after authorized treatment, and it is also the residue obtained following filtration or centrifugation [6]. Lees contain dead yeast, yeast residues, and particles generated from grapes or generated as a result of the chemical/microbiological changes that normally precipitate at the bottom of wine vessels [7]. Currently, this waste material is often dumped in landfills or spread in vineyards as compost, which was reported to have a negative impact on the environment and soil [8]. Therefore, a cost-effective and environmentally friendly way of using this material should be of interest to the industry. Previously, Alonso et al. [9] reported that wine lees have a high phenolic content and exhibit strong antioxidant activity. Furthermore, Hwang et al. [7] found that ice cream’s antioxidant properties were improved with the addition of wine lees.

It is expected that wine lees produced commercially will have different chemical compositions and characteristics due to the fact that lees can be obtained from a range of vinification techniques due to differences in the maceration time, yeast strain, time in oak barrels, malolactic fermentation, and vast grape varieties used in the wine industry. It is reasonable to suggest that different management solutions and the potential usage of wine lees can be devised based on their chemical compositions and characteristics. However, such information is not available, and research is required to benchmark the effects of these factors. To maximize the potential use of this by-product, it is necessary to characterize a wide range of commercial lees. The present study examined the phenolics characteristics of lees from various sources, utilizing a range of the winemaking techniques of two grape varieties to improve the understanding of this material and to devise useful strategies to add value to this waste stream.

## 2. Materials and Methods

### 2.1. Wine Lees Sample Preparation

Wine lees used in this study were kindly provided by Pegasus Bay Winery (Waipara, Canterbury), Omihi Hills Winery (Waipara, Canterbury), Felton Road Winery (Bannockburn, Central Otago), and Vinpro Winery (Cromwell, Central Otago) of New Zealand between April and August of the wine production season (Table 1). A total of sixteen lees samples (10 kg each in different batches) from commercial winemaking were analyzed and presented in the present study, including 10 Pinot Noir (PN) and 6 Riesling (RL) samples. The sample information is shown in Table 1. The vinification techniques are discussed in the Results Section (Section 3) and Discussion Section (Section 4).

Frozen lees samples were freeze-dried (VirTis Freezemobile 12SL, New York, NY, USA) at a pressure of 0.5 mbar. Freeze-dried (FD) lees samples were then homogenized with a mortar and pestle and kept in air-tight containers at −20 °C. The lees samples were extracted as described by Mercurio et al. [10]. A two-step extraction was conducted. Five mL of acidified 50% (containing 1% HCl) ethanol was added to 0.5 g FD PN samples as well as to 3 g FD RL samples. The samples were then shaken using a thermostatic orbital shaker (Model OM11, Ratek Instruments Ltd., Boronia, VIC, Australia). Samples were centrifuged at 2890× *g* for 5 min using a Megafuge 1.0 with a Siehe rotor (Model TS-HM 10, Thermo Fisher Scientific, waltham, MA, USA). The second step of the extraction repeated the procedure above. Supernatants from each step of extraction of the subsampled lees samples were decanted, mixed, and stored at −20 °C. Wine lees and sample preparation were done in triplicate.

### 2.2. Chemicals

Methyl cellulose, gallic acid, and resveratrol were purchased from Sigma Chemical Co. (St. Louis, MO, USA). Folin–Ciocalteu reagent; α, α-diphenyl-β-picrylhydrazyl (DPPH); 2, 2′-azobis (2-amidinopropane) dihydrochloride (AAPH); catechin and epigallocatechin gallate; malvidin; caftaric acid; cinnamic acid; and quercetin were obtained from Sigma–Aldrich Chemical Co. (Steinheim, Germany). Hydroxybenzoic acid was purchased from Fisons Scientific Apparatus (London, UK). Ethanol (100%) was obtained from Fisher Scientific (Loughborough, UK). Methanol (100%) and hydrochloride were obtained from Merck (Darmstadt, Germany). All reagents and chemicals used in this study were of analytical grade or higher.

### 2.3. Determination of Total Phenol and Total Tannins

The total phenol content (TPC) of wine lees extracts was determined using Folin–Ciocalteu reagent as described by Makkar et al. [11]. Gallic acid standards in the range of 50–1000 mg/L were used to plot standard curve. The total phenolic content was expressed as mg of gallic acid equivalents (GAE) per gram of dry lees (mg GAE/g DM).

The total tannin content (TTC) of extracts of FD wine lees was determined using the methylcellulose precipitable (MCP) tannin assay as described by Sarneckis et al. [12]. Epicatechin standards at concentrations of 5, 25, 50, 75 and 100 µg/mL were used for plotting standard curve. Results were reported on epicatechin equivalent basis (mg epicatechin eq/g DM).

### 2.4. Quantification of Anthocyanins

The total monomeric anthocyanin content of extracts of FD wine lees was determined using the pH differential method as described by Giusti and Wrolstad [13] with modification. Two dilutions of each sample were prepared by adding 1350 μL of potassium chloride (pH 1.0) and sodium acetate buffer (pH 4.5) to 150 μL of extracts, respectively. The prepared dilutions were left for 15 min at room temperature for equilibration. The dilutions (at pH 1.0 and 4.5) were then measured by spectrophotometer at 520 nm (A _λvis-max_) and 700 nm (A _700_) against water blank. Monomeric anthocyanin pigments can be calculated using the following equations:

A = (A _λvis-max_ − A _700_) _pH 1.0_ − A _λvis-max_ − A _700_) _pH 4.5_ (1)

Monomeric anthocyanin pigments (mg/L) = (A × MW × DF × 1000)/ϵ(2)

where A is the absorbance of the diluted sample, MW is the molecular weight (511.2 g/mol), DF is the dilution factor (DF = 10), ϵ is the molar absorptivity (ϵ = 28,000 of Mavidin-3-glucoside was used in the current study), A _λvis-max_ = 520 nm, and A _700_ = 700 nm.

For the determination of polymeric anthocyanin, the method described by Giusti and Wrolstad [13] was adopted with modification, where 200 g/L potassium metabisulfite solution and 0.025 M potassium chloride buffer (pH 3.5) were used. The samples were firstly diluted 30 times with 0.025 M potassium chloride buffer followed by transferring 2.8 mL of diluted samples to cuvettes. Then 0.2 mL of bisulfite solution was added to the cuvettes with test samples, and the same volume of deionized water was added to the control samples. The prepared samples were left for 15 min at room temperature for equilibration, and then the absorbance was measured using a spectrophotometer (UV-1800, Shimadzu Corporation, Tokyo, Japan) at 420 nm (A _420_), 520 nm (A _λvis-max_), and 700 nm (A _700_) against water blank. The percentage of polymeric anthocyanin was calculated as written below:

Color density = [(A _420_ − A _700_) + (A _λvis-max_ − A _700_)] × DF (3)

The calculation is based on the absorbance of the control sample, and DF = 30 in the current study.

Polymeric color = [(A _420_ − A _700_) + (A _λvis-max_ − A _700_)] × DF (4)

The calculation is based on the absorbance of the treatment sample, and DF = 30 in the current study.

Percentage polymeric anthocyanin = polymeric color/color density × 100(5)

### 2.5. Characterization of Phenolic Profile

Phenolic compounds were determined using the method reported by Kemp et al. [14]. Wine lees and model wine extracts were injected (10 µL) into an LCMS system (Shimadzu 2010, Japan) equipped with a PDA detector (D2 and W Lamp 200–600 nm) and a Grace Davison C18 column (250 × 2.1, particle size 5µm, held at 25 °C). The flow rate was kept at 0.5 mL/min throughout running time. All samples were filtered using a 0.45 µm PTFE filter (2165 Catalogue, Grace Davison) before injection. The analysis was carried out with electrospray ionization (ESI) interface, and the MS parameters were a detector voltage of 1.5 kV, an interface voltage of 4.5 kV, a curved desolvation line of −45 V, an array reflector voltage of 150 V, and heat block and CDL temperatures that were kept at 200 and 250 °C, respectively.

Phenolic compounds in the samples were confirmed individually by mass spectroscopy and were quantified with a PDA detector at 280 nm using corresponding standards.

### 2.6. Determination of Antioxidant Activities

Antioxidant activity of wine lees extracts was determined using α, α-diphenyl-β-picrylhydrazyl radical (DPPH•) and oxygen radical antioxidant capacity (ORAC) methods.

The ability of lees extracts to scavenge the DPPH• was evaluated using method previously described by Sánchez-Moreno et al. [15] with modification. Each lees extract was serially diluted using 50% ethanol solution to produce concentrations replacing 50, 25, 12.5, 6.25, 3.13, and 1.56% of the original extracts. A 75 μL aliquot of each diluted sample was added to 2925 μL of 0.025 g/L DPPH• solution (prepared in 100% HPLC Grade methanol) in a 3 mL visible cuvette and was mixed by gentle inversion. DPPH• solutions were prepared daily and kept on ice in a light-tight container. The absorbance at 515 nm (A _515_) was measured at 0, 5, 10, 20, 30, and 60 min of reaction using a spectrophotometer against a water blank. The cuvettes were covered with parafilm and kept in a light-tight container between absorbance measurements.

The concentration of remaining DPPH• in the reaction medium at each reaction time was calculated from a calibration curve of DPPH• with absorbance at 515 nm (A _515_) against concentration. The equation below was applied to calculate the percentage of remaining DPPH• at the reaction time of 10 min. The percentage of remaining DPPH• was then plotted against the weight (mg) of extract.

% DPPH•_R_ = [(DPPH•) _T_/(DPPH•) _T=0_] × 100 (6)

where (DPPH•) _T_ is the DPPH• concentration at a plateau; (DPPH•) _T=0_ is the DPPH• concentration at zero reaction time.

Results were also presented as EC_50_ (effective concentration, or the amount of sample required to decrease the initial concentration of DPPH• by 50%). EC_50_ of different sample extracts were calculated from the regression equation.

The ORAC assay uses 2,2′-azobis (2-amidinopropane) dihydrochloride (AAPH) as a peroxyl radical generator [16] and reports the antioxidant activity of a compound relative to a standard antioxidant (Trolox). The antioxidant capacity of lees extracts was determined using the ORAC assay according to Huang et al. [17] with modifications. All determinations were performed on three subsamples, and the measurements were in triplicate. FLUOstar Omega multifunctional microplate reader (BMG LABTECH, Ortenberg, Germany) was used to measure the fluorescein intensity. The instrument was set up to read fluorescence intensity mode. The excitation, emission, and cut-off wavelengths were 485 nm, 538 nm, and 530 nm, respectively, and the gain was adjusted to 85%. The measurement was taken over 60 min at 1 min intervals.

Diluted antioxidant standard and samples (25 μL) were added to the 96-well microplate followed by the addition of 150 μL of 10 nM fresh fluorescein working solution to each well. The microplate was then covered by parafilm and incubated at 37 °C for 30 min. After incubation, 25 μL of the AAPH (65 mg/mL) solution was automatically injected into each well by the plate reader pump followed by 5 s of shaking.

The area-under-the-curve (*AUC*) method was used for the quantification of the ORAC [17]. *AUC*s of blank and of antioxidant were calculated from the equation below:

(7) $$AUC=1+\overset{60}{\underset{i=1}{\sum }}\frac{RFUt=i}{RFU0}$$

where *RFU*0 is the relative fluorescence value of time point zero and *RFUt* is relative fluorescence value of time points. The net *AUC* can be calculated by subtracting *AUC* (blank) from *AUC* (antioxidant). Based on the standard concentration between 0 and 0.05 mg/mL Trolox, the antioxidant activity was calculated and expressed in equivalent Trolox concentration (TE).

### 2.7. Determination of Antimicrobial Activity

The antimicrobial and antifungal activities of the wine lees extracts were determined by conducting the minimum inhibitory concentration (MIC) assay in microtiter plates with the nutrient broth. Bacteria (Gram-positive *S. aureus* and Gram-negative. *E. coli*) and fungi (*C. albicans*) were incubated in Mueller–Hinton broth (MHB) and Sabouraud dextrose broth (SAB), respectively, as described by Wood and Washington [18].

The cultures were then centrifuged at 3000 rpm (Beckman J2–21M/E centrifuge, JA-14 rotor, Beckman Coulter Inc., Fullerton, CA, USA) for 20 min and diluted with 0.1% peptone water with reference to the viable counts, and they were photometrically adjusted to achieve an inoculum size of approximately 10^7^ CFU/mL. The preparation of the inoculum was completed within 30 min to minimize the reproduction of microorganisms which may lead to changes in the density.

To perform a minimum inhibitory concentration assay, 100 µL of nutrient broth was firstly pipetted into each well of a microtiter plate. A total of 100 µL of wine lees extracts previously filtered through 0.45 µm filters were pipetted into the 2nd column wells, and serial dilutions were made in the wells along the microtiter plate at 50%, 25%, 12.5%, 6.25%, 3.125%, and 1.5625%. A total of 10 µL of the microorganism was added to each well, and ampicillin and amphotericin B were used as standard antibacterial and antifungal agents, respectively. Stock solutions of antimicrobial agents were prepared at a concentration of 0.8 mg/mL (*w*/*v*) [19].

The MIC values were determined using a microplate reader (BioTek^®^ Synergy 2, Gen 5^TM^, Winooski, VT, USA) after 24 h of incubation of the mixture at 37 °C for *E. coli* and *S. aureus* and after 48 h for *C. albicans*. Absorbance readings using the microplate reader were set at 600 nm and at 37 °C. All measurements of MIC_50_ values were performed in triplicate, including three controls with 50% acidified ethanol, MHB, and microorganisms.

### 2.8. Statistical Analysis

Analysis of variance (ANOVA) was carried out to investigate the effects of samples (dependent variable) on the measured parameters. Significant difference at *p*-value < 0.05 was tested by application of Tukey’s multiple comparisons test. Error bars in graphs indicate the standard deviation (SD) of the mean. Pearson correlation coefficients, among the measured parameters, were also calculated. Genstat^®^ software (Version 9.0, VSNI, Hemel Hempstead, UK) was used for statistical analysis.

## 3. Results

### 3.1. Total Phenolic Content and Total Tannin Content

**Figure 1:**
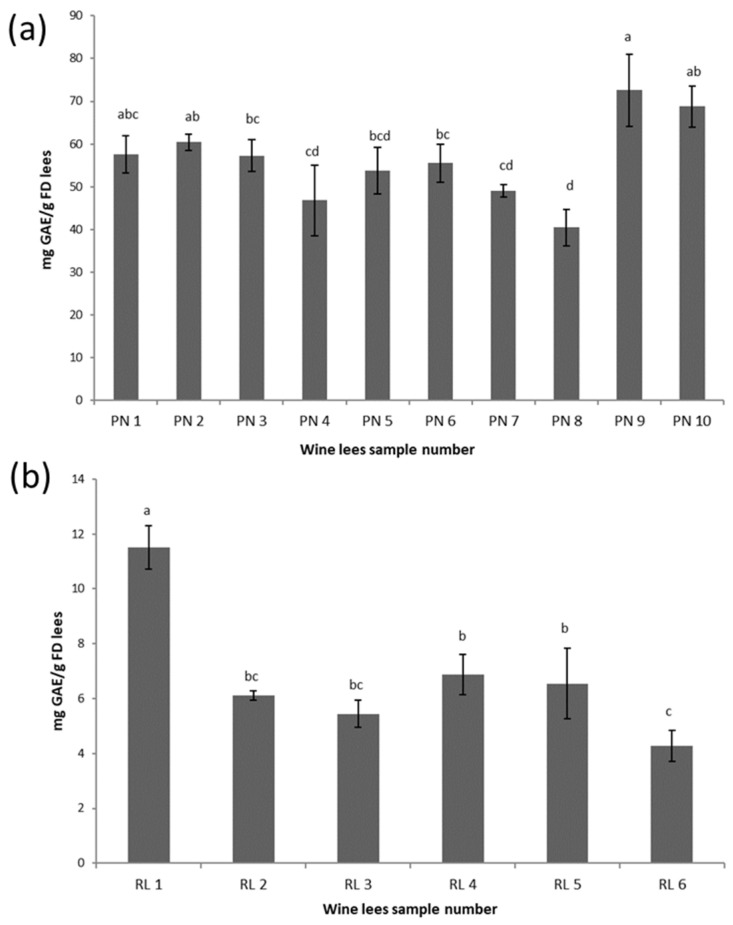
Total phenolic content (mg GAE/g DM lees) of PN (**a**) and RL (**b**) wine lees extracts. Samples that do not share the same letter (a–d) are significantly different (*p* < 0.05). Error bars represent the standard deviation of replicate samples (*n* = 3).

Lower TPC values were observed in RL lees extracts compared to PN lees extracts (Figure 1). The TPC values of different RL lees extracts in the current study were not significantly different (*p* > 0.05). This might have been caused by the combined statistical analysis of PN and RL samples which generated a high pooled standard error of the means and masked the small differences within the RL samples. Although not statistically significant, the highest TPC value of 11.5 ± 0.8 mg GAE/g DM lees was observed in RL1 lees extracts, and this might be attributed to the application of pre-fermentation maceration which allowed the contact of grape skin with the must.

Within the Pinot Noir lees samples, PN9 and PN10 had the highest TPC values at 72.6 ± 8.4 and 68.7 ± 4.9 mg GAE/g DM lees, respectively (Figure 1). This high level of the TPC may be attributed to the different winemaking stages that the lees samples were collected at. The PN9 and PN10 samples were collected post alcoholic fermentation in May, which may have contained fine residues from the pressing of the marc (grape seeds, skins, and pulps). Pinot Noir lees samples PN1 to PN6 were collected much later when there would be little likelihood of the samples containing any marc materials as a result of a series of progressive racking post alcoholic fermentation. The lowest TPC value was observed in PN4 and PN8 (46.8 ± 8.3 and 40.4 ± 5.5 mg GAE/g DM lees). The reason for the low TPC values in these samples appeared to be that they were obtained from wines that underwent carbonic maceration, where intact grapes are placed in a closed tank under a carbon-dioxide-rich atmosphere. The proportions of the whole berry content in PN4 and PN8 ferments were 100%, which meant less phenolic compounds were extracted to must during maceration compared with normal maceration with crushed grapes. PN7 was obtained in late April; however, no significant differences were found among the TPC values of the rest (PN1, PN2, PN3 PN5, PN6, and PN7) of the PN lees extracts (*p* > 0.05). This may also indicate that the interaction of several factors, such as machine and hand harvest, the fermentation temperature, and pre- and post- fermentation maceration, can remove any significant impact of any one single factor on the TPC of PN lees.

Cheng [19] reported the TPC of the seed and skin of five white and two red varieties. The TPC of the white grape seeds and skin ranged from 350.0 to 440.5 and from 8.0 to 20.1 mg GAE/g DM extracts, respectively. To compare the results from the current study with the published results, the unit was converted from mg GAE/g FD lees to mg GAE/g DM extracts. The TPC of the RL lees in the current study ranged from 39. 4 ± 1.2 to 239.4 ± 16.6 mg GAE/g DM extracts which were higher than the TPC of the white grape skins but less than that of the grape seeds. The TPC that Cheng [19] observed in the red grape seeds and skins ranged from 350.7 to 420.8 and from 10.4 to 20.6 mg GAE/g DM extracts, respectively. In comparison, the TPC of the PN lees (ranged from 104. 6 ± 18.8 to 291.1 ± 33.7 mg GAE/g DM extracts) was greater than that of the red skins but less than that of the seeds. Tao et al. [20] reported that the ultrasound-assisted extraction of phenolics with yield optimized conditions (43.9% ethanol, 60:1 *w*/*v*, 60 °C, 25 min) from wine lees was 58.76 mg GAE/g. In contrast, a higher yield was obtained in the current study with 50% acidified ethanol. Thus, the increasing TPC in wastes generated during winemaking seemed to occur in the following order: skin < lees < seeds.

**Figure 2:**
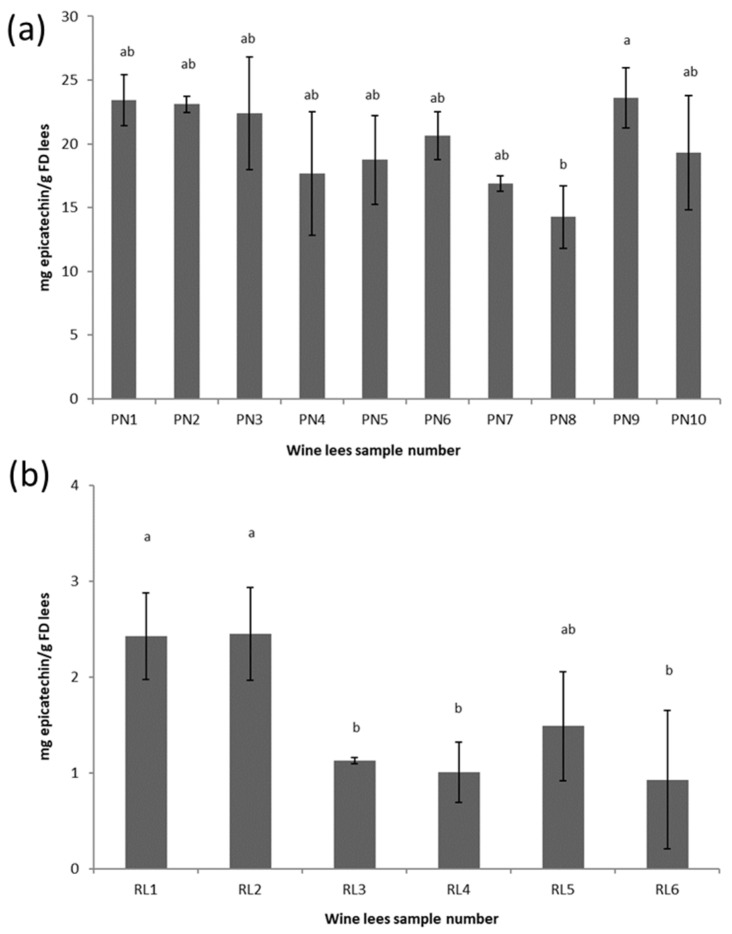
Total tannin content (mg epicatechin equivalent/g DM lees) of PN (**a**) and RL (**b**) dry-matter lees. Means that do not share the same letter (a,c) are significantly different (*p* < 0.05). Error bars represent the standard deviation of replicate samples (*n* = 3).

The pattern of the TTC was similar compared to the TPC, and a higher (*p* < 0.05) TTC was found in the PN lees compared to the RL lees (Figure 2). The TTC of the PN8 sample was the lowest, which was consistent with the TPC of the same sample. Vinification techniques, including additions of oenological tannins and pectolytic enzymes, were applied in PN8 sample processing to maximize the phenolic and tannin extractions, which might indicate that the initial grape quality of PN8 was poor in terms of tannins.

Ye et al. [21] characterized the phenolic compounds in Chardonnay, Sauvignon Blanc, Pinot Gris, Pinot Noir, and Rosé wine lees. Wine lees can be divided into low-, middle-, and high-phenolic-content materials according to the TPC and the TTC. The highest TPC and TTC were observed in the Pinot Noir lees (17–40 mg GAE/g DM lees and 10–20 mg epicatechin/g DM lees), and the lowest ones were found in the white wine lees (3–10 mg GAE/g DM lees and 1–5 mg epicatechin/g DM lees). This is consistent with the findings in the current study. In addition, Ye et al. [21] also pointed out that the TPC in wine wastes can also be affected by the vinification techniques used, such as the fermentation temperature, maceration, pump over, the use of oak barrels, etc.

### 3.2. Quantification of Anthocyanins

**Figure 3:**
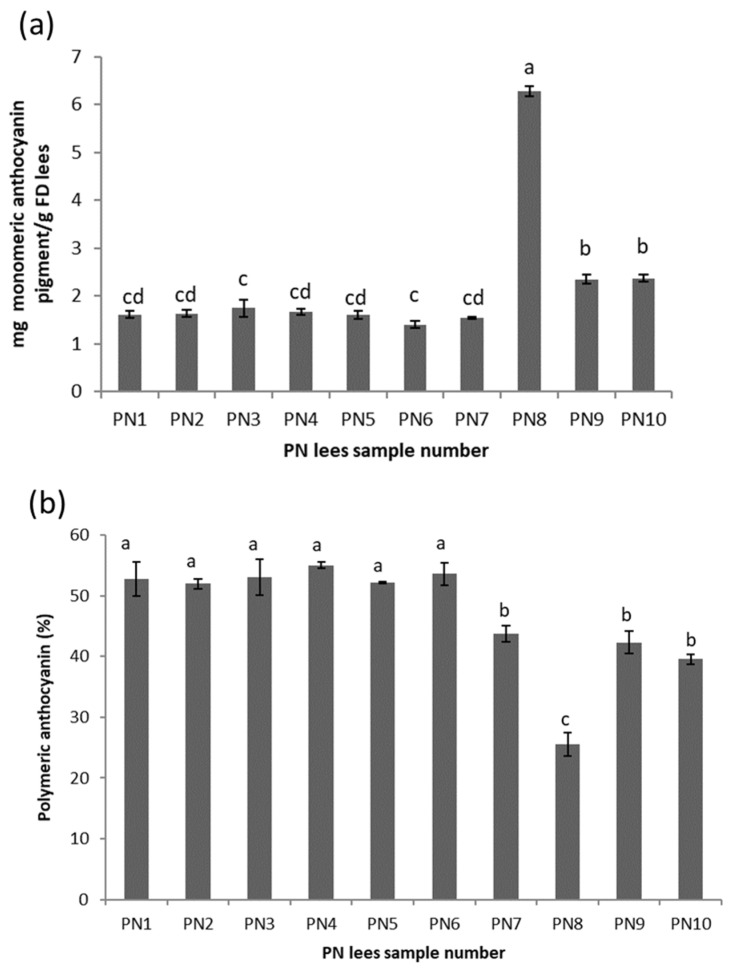
(**a**) Total monomeric anthocyanin pigment (mg of monomeric anthocyanin pigment/g of FD lees) of different wine lees extracts. Means that do not share the same letter (a–d) are significantly different (*p* < 0.05). Error bars represent the standard deviation of replicate samples (*n* = 3). (**b**) Polymeric anthocyanin pigment percentage of different wine lees extracts investigated in the present study.

For *Vitis vinifera* varieties, anthocyanins are localized in grape skins and are extracted by maceration in the fermenting must. The total monomeric anthocyanin contents (T-mAcy) of sixteen wine lees extracts were measured, and anthocyanins were only detected in the PN lees extracts. The T-mAcy of the ten PN lees extracts is shown in Figure 3a. The PN lees extracts can be divided into the following three groups with an increasing order: PN1 to PN7, PN9 to PN 10, and PN8. The lowest T-mAcy values were found in the extracts of PN1 to PN7, which ranged from 1.4 ± 0.1 to 1.8 ± 0.2 mg/g DM lees. Within this group of samples, the T-mAcys were very similar, possibly due to the PN1 to PN6 lees samples being obtained from the same vineyard and having similar vinification techniques. PN7 showed a similar T-mAcy to that of the samples in the first group although PN7 was obtained about 3 months earlier than PN1 to PN6. The low T-mAcy found in PN7 may be explained by the proportion of the whole berry content (30%) that remained uncrushed during fermentation, which restricted the extraction of monomeric anthocyanin to the must. PN9 and PN10 samples (collected at the end of alcoholic fermentation without malolactic fermentation) had a higher T-mAcy than the first group, with T-mAcy values of 2.4 ± 0.1 mg/g DM lees. The highest T-mAcy was found in PN8 (6.3 ± 0.1 mg/g FD lees), which might be attributed to the addition of 20 mg/L of the pectolytic enzyme. The application of the pectolytic enzyme in winemaking is to augment anthocyanin liberation and to improve the wine color by breaking down the cell walls of the grape skins [22].

The percentage of the polymeric anthocyanin (pAcy%) in the ten PN lees extracts is shown in Figure 3b. The results can be divided into three groups: high (PN1 to PN6), medium (PN7, PN9, and PN10), and low (PN8). The pAcy% in PN1 to PN6 ranged from 52.0 ± 0.8 to 55.0 ± 0.5%. These six lees samples were obtained in July and August post malolactic fermentation (MLF), which was about 2 to 3 months later than when PN7 to PN10 were obtained. These results were inconsistent with the finding in a wine reported by Burns and Osborne [23], where a wine that underwent MLF showed a significantly lower polymeric pigment content than wines that had not undergone MLF. The possible explanation for why PN1 to PN6 showed a higher pAcy% could be the polymerized products formed from anthocyanins in the course of wine aging, often described as polymeric pigments, resulting from the reaction of anthocyanins with tannins [24]. It was then suspected that PN1 to PN6 underwent more polymerization during the extra 2 to 3 months before taking their lees sample.

### 3.3. Characterization of Phenolic Profile

The identification and quantification of the ten Pinot Noir (PN) lees and the six Riesling (RL) lees are shown in Table 2.

For the PN lees, the gallic acid concentrations ranged from 0.5 to 2.2 mg/g extracts. Gallic acid was the predominant nonflavonoid in PN1 to PN6, which was higher than the total hydroxycinnamic acids measured in the study. In contrast, the gallic acid concentrations were lower than those of the hydroxycinnamic acids in PN7, PN9, and PN10. After fermentation and pressing, PN1 to PN6 were transferred to oak barrels. Oak cooperage has been noted as a source of phenolic acids [25]. For these samples, it was likely that gallic acid was released from the oak barrels during barrel aging, which was in contrast to PN7, PN9, and PN10 which utilized stainless steel tanks. The only exception was PN8 which showed a lower gallic acid content than that of hydroxycinnamic acids after being aged in barrels. The chemical composition of oak barrels varies by their origin, seasoning, toasting, and production technique [25]. However, the type and age of the barrel were not investigated in the current study.

Flavan-3-ols were the primary flavonoids which were consistent with the composition of grape seeds reported by Bakkalbaşı et al. [26]. The content of catechin was found to be the highest in the PN lees, which ranged from 0.2 to 5.9 mg/g extract, followed by that of epicatechin (ranged from 0.9 to 2. 9 mg/g extract) and total procyanidins (0.7 to 4. 1 mg/g extract). In comparison, the PN lees showed a lower content of catechin and epicatechin than the grape seeds [26]; however, more types of flavan-3-ol were identified in the lees than in the grape skins for which only catechin and procyanidin B were detected [27].

For the RL lees, the derivatives of hydroxycinnamic acids were the primary nonflavonoid in the lees. White wines are normally stored in stainless steel tanks; thus, there are no contributions of phenolic acids from oak extraction. Additionally, hydroxycinnamic acids are largely found in the flesh of the berries and are readily released upon crushing and pressing, while hydroxybenzoic acids exist in the skins and seeds of the grape berries [28]. In general, the flavan-3-ols were found to be lower in most of the RL lees when compare with the PN lees (*p* < 0.05). This can be attributed to the vinification of white wine in which the contact of skins and seeds with must is avoided. This was consistent with the observation reported in an earlier section of the current study.

### 3.4. Determination of Antioxidant Activities

The EC_50_ values of the lees extracts were determined, and extracts of the PN lees exhibited significantly lower EC_50_ values (from 9.9 ± 1.7 to 17.3 ± 0.1 g FD lees/g DPPH) than those of the RL lees (from 107.3 ± 8.3 to 251.9 ± 13.2 g FD lees/g DPPH) (*p* < 0.05) (Table 3). These results were consistent with those found for the TPC and the TTC in general. Among the RL extracts, RL1 and RL3 showed the lowest (107.3 ± 8.3 g DM lees/g DPPH) and highest (251.9 ± 13.2 g DM lees/g DPPH) EC_50_ values, respectively. RL1 was machine-harvested and underwent pre-maceration before fermentation, which is occasionally practiced on white grapes to enhance the varietal characteristics. Olejar et al. [29] reported that Sauvignon Blanc that was made from machine-harvested grapes had a significantly higher amount of gallic acid, coutaric acid, caffeic acid, ferulic acid, resveratrol, and epicatechin. When mechanical harvesting was accompanied with pre-maceration, the resulting wine showed an increased phenolic content and antioxidant activity with statistical significance [29]. 

Cheng [19] reported the radical scavenging activity (EC_50_) of Pinot Noir by-products: The seeds (156.8 mg extract/g DPPH) exhibited the highest antioxidant activity followed by the pomace (304.02 mg extract/g DPPH) and skins (1365.7 mg extract/g DPPH) (seeds > pomace > skin). The EC_50_ values of the RL seeds and skins were 1070 and 22451 mg extract/g DPPH, respectively [19]. In contrast, the EC_50_ values of the PN lees observed in the current study were in the range of 3530 to 7200 mg extract/g DPPH, which indicated a lower antioxidant activity than that of the PN seeds, pomace, and skins. Similar results were also found with the RL lees.

Researchers normally use more than one method for the analysis and comparison of the free radical scavenging ability because different methods target different compounds. The results of the ORAC clearly distinguished the differences in the free radical scavenging activity between the white and red wine lees, which was consistent with the observation in the DPPH assay (Table 3). The results showed strong correlations between DPPH, the TPC, the TTC, and the ORAC (Table 4). Such a correlation was consistent with other findings in red wine [30]. However, some ORAC results were slightly different from those observed with the DPPH assay; for example, contrary results were found in the PN7 and PN8 samples. Such observations confirmed the suggestion that was made earlier that the DPPH and ORAC assays target different groups of compounds with respect to the free radical scavenging activity.

**Figure 4:**
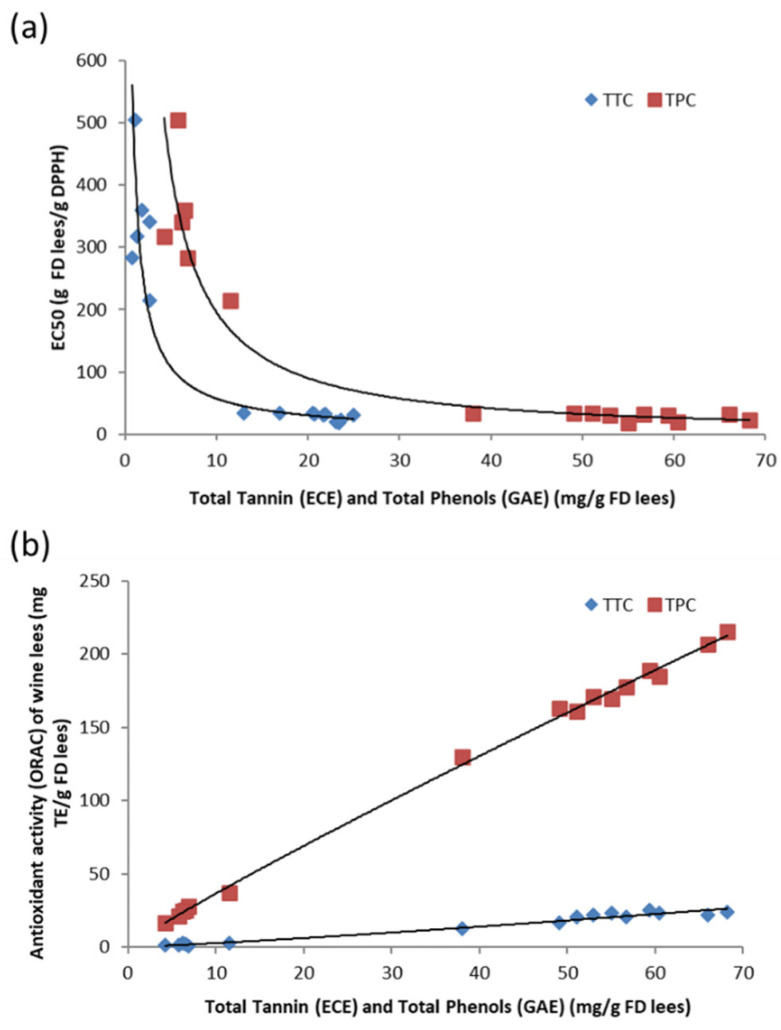
(**a**). The relationship between EC_50_ of wine lees extracts and their TTCs and TPCs; (**b**). The relationship between oxygen radical antioxidant capacity (ORAC) of wine lees extracts and their TTCs and TPCs. ECE represents epicatechin equivalent and GAE represents gallic acid equivalent.

The relationship between the DPPH antioxidant activity (EC_50_) and the TPC and TTC of the sixteen wine lees extracts is shown in Figure 4a. The DPPH EC_50_ were negatively correlated with the TPC and TTC of wine lees extracts. The results of the ORAC were positively correlated with the TPC and TTC of wine lees extracts (Figure 4b). The results were easily divided into three groups: low (RL lees, approximately 16.6 to 37.3 mg TE/g FD lees), medium (PN8, 130.0 mg TE/g FD lees), and high (PNs, > 130.0 mg TE/g FD lees). The ORAC assay was strongly correlated to the TPC and TTC, indicating that most of the antioxidant activities observed were related to the TPC and TTC of lees extracts. In addition, there were linear relationships between the antioxidant activities and the TPC and TTC in which both the TPC and TTC were proportional to antioxidant activity (ORAC). It is worth noting that tannins appear to contribute to about one third of the activity (Figure 4b).

### 3.5. Determination of Antimicrobial Activities

The MIC values of the lees extracts ranged from 0.78 mg/mL to 50.00 mg/mL (Table 5). Both the PN and RL lees extracts had inhibitory effects on the growth of *S. aureus*. The minimum inhibitory concentration varied among the lees samples. The MIC value of Gram-positive *S. aureus* ranged from 0.78 mg/mL (RL5) to 6.25 mg/mL, which was the strongest antimicrobial activity found in the RL lees extracts.

Growth inhibition was also observed in *E. coli.* All of the white and three of the red (PN3, PN6, and PN8) lees extracts exhibited antibacterial effects. The MIC values for Gram-negative *E. coli* ranged from 3.13 to 25.00 mg/mL.

The PN lees extracts did not have any antifungal activity against *C. albicans*. *C. albicans* appeared to be more resistant to the lees extracts, and only RL1, RL2, and RL4 inhibited its growth at concentrations that ranged from 12.50 to 50.00 mg/mL. The RL5 lees extracts were found to be the most effective in inhibiting the growth of *S. aureus* and *E. coli*, but these extracts had no antifungal activity against *C. albicans*, indicating that bacteria and fungi exhibit different resistance abilities to bioactive compounds in lees extracts.

The PN lees extracts might have lower antimicrobial and antifungal activities when compared with the seed, skin, and pomace extracts from the same grape variety. Cheng et al. [31] investigated the antimicrobial and antifungal activities of Pinot Noir (PN) seed, skin, and pomace. For example, PN seed, skin, and pomace extracts inhibited the growth of *S. aureus* and *C. albicans* at concentrations that ranged from 0.39 to 0.78 mg/mL. A similar trend was observed in which PN seed, skin, and pomace extracts were less effective against *E. coli*. In comparison with a Merlot pomace extract [32], PN lees extracts showed a higher antimicrobial activity against *S. aureus* (7.81 mg/mL) and *E. coli* (62.5 mg/mL).

The antimicrobial activity of plant material extracts in previous studies has been classified as strong (MIC: <500 µg/mL), medium (MIC: 600–1500 µg/mL), and weak (MIC: >1600 µg/mL) [33,34,35,36]. According to this system, the PN and RL lees extracts could be regarded as weak inhibitors against *S. aureus* and *E. coli*. The RL5 extract showed medium inhibition effects on *S. aureus*.

## 4. Discussion

The TPC of the PN lees extracts was significantly higher (*p* < 0.05) than that of the RL lees extracts (Figure 1). The main reason for this variation can be attributed to the differences in the vinification process during winemaking. During the making of red wine, maceration is conducted before pressing, which facilitates the extraction of nutrients, flavorants, and other constituents from the pulp, skins, seeds, and occasionally the stems [25]. In this study, all 10 of the PN samples received maceration and were fermented with skin contact, which allowed the extraction of phenolic compounds. In addition, increased phenolic extraction occurred due to a change in the solvent from aqueous to hydroalcoholic, which facilitated the transfer of phenolics from the seeds and skin to the wine. In white winemaking (such as RL), the contact between the grape juice and the skins and seeds is normally either avoided altogether (RL2 to RL6) or occurs for only a short period of time (RL1) and, therefore, lower amounts of phenolics are extracted.

On the other hand, even when white grapes are fermented, such as a typical red wine in the presence of skins and seeds, the apparent difference in the phenolic compositions of the pomace-fermented white wines and red wines is the anthocyanin content [37]. The presence of anthocyanins is believed to be able to enhance the extraction from the skins and seeds due to a higher solubility of the extracted phenols caused by the increased polarity of the anthocyanin-associated complexes [38,39].

Lees material contains dead yeast, yeast residues, and particles precipitated at the bottom of wine vessels [7]. Mannoproteins are the basic components that make up most of the external surfaces of yeast cells, and these macromolecules provide binding sites for the adsorption of phenols [40]. Therefore, the phenolics in wine can then be adsorbed on the surface of yeast and can be precipitated with lees [41]. Aksu and Dönmez [42] studied the biosorption characteristics of yeast cells and suggested that the availability of binding sites was the main factor limiting the adsorption of phenols.

Consequently, the method of extraction and the extraction ability (e.g., the presence of ethanol and anthocyanin) lead to varying amounts of phenolics being extracted into the wine. It would be expected that the vinification methods that lead to more phenolic extraction into wine or juice would potentially result in higher concentrations of phenolic compounds in lees. This was evident since the use of carbonic maceration (PN4 and PN8), the degree of crushing, and the greater proportion of the whole berry content remaining during red wine fermentation (PN7) limited the extraction of various phenolic compounds compared with the rest of the PN lees samples and since the RL lees have significantly lower phenolic compounds compared to the PN lees.

Polymerization will naturally occur given that there is a sufficient amount of phenols present. For example, the highest level of T-mAcys was observed in PN8, which also had the lowest level of pAcy%, TTC, and TPC (Figure 1, Figure 2 and Figure 3), indicating that the process of polymerization may not progress well due to the lack of tannins and phenolics. On the other hand, the lees with high phenolic and tannin contents can be used for removing excessive color (anthocyanin) from wine due to the physicochemical adsorption of anthocyanin. For example, Pinot Noir is one of the major grape varieties used for making Champagne. The application of PN lees can remove the excessive anthocyanin that diffused during the pressing of overmature or botrytized grapes [41].

Among PN extracts, although not statistically significant, PN1 and PN2 showed the lowest EC_50_ values and, hence, the highest antioxidant activity measured by the DPPH assay (Table 3), which might be attributed to hand harvesting and the addition of SO_2_ during grape processing. Arfelli et al. [43] reported lower phenolic content in the wine made from grapes that were mechanically harvested due to the berry breakage and the occurrences of oxidation; the total phenol content in the must was higher when SO_2_ and oenological tannins were added.

It is interesting to point out that PN8 possessed the lowest TPC and TTC but showed a similar antioxidant activity (DPPH) to the rest of the PN extracts. This was in agreement with a previous study that reported that the correlation between the TPC and antioxidant activity will vary due to the diverse quantifying methods applied to evaluate the antioxidant activity and due to the complexity of the natural mixture of the phenolic compounds [44]. Therefore, not all antioxidant activities correlate positively with the TPC. In addition, PN8 also received the addition of oenological tannins during maceration. Oenological tannins are commercial tannins produced by the extraction of tannins from oak, chestnut, or birch woods, and other suitable plant sources [45], which consist of proanthocyanidins and ellagic tannins. Moreover, gallic acid is the basic unit of ellagic acid which is reported to be a strong antioxidant with an EC_50_ value of 0.026 g/g DPPH [46,47].

As characterized by HPLC, it was suggested that gallic acid was the predominant nonflavonoid in PN1 to PN6 (Table 2). PN9 and PN10 were of the highest TPC values with lower antioxidant activities (indicated by higher EC_50_ values) than the PNs with lower TPC values. PN9 and PN10 were processed in stainless steel tanks, which resulted in higher hydroxycinnamic acids (especially caftaric acid) compared to gallic acid (Table 2). In contrast, PN1 to PN6 were high in gallic acid which indicates that gallic acid is important for DPPH scavenging while hydroxycinnamic acids may not be as important. On the other hand, caftaric acid, which was the predominate hydroxycinnamic acid in the PN lees (Table 2), was previously reported to have a three times higher EC_50_ value than gallic acid [47], indicating its lower antioxidant activity.

The contribution of the different phenolic compounds to the antioxidant activities had been investigated in previous studies. Flavanols (especially catechin) were found to be negatively correlated with the ORAC assay but positively correlated with the FRAP assay (ferric reducing ability of plasma); quercetin-3-O-glucuronide was found to be positively correlated to the ORAC and HOSC (hydroxyl radical averting capacity) [48]. Bekhit et al. [44] measured the antioxidant activity of the grape skin tea infusion and demonstrated that the tea infusion with high antioxidant activities (SASA and DPPH) contained a higher level of epicatechin, epicatechin gallate, gallocatechin, epigallocatechin, and gallic acid. In addition, Ky et al. [49] investigated the correlation between different antioxidant assays and the total proanthocyanidin content in grapes and pomace and found that monomers and dimers had a strong correlation with the DPPH, ABTS, and FRAP assays. However, none of the above studies investigated the correlation between phenolic compounds extracted from wine lees and antioxidant activities.

The majority of the free radical scavenging activity of wine by-products comes from phenolic compounds remaining after extraction (e.g., crushing, increased temperature, maceration, and pump over/punch down) during the winemaking process. This is in agreement with Jara-Palacios’ [48] study which reported that the antioxidant activity of the wine wastes is usually directly related to the concentration of phenolic compounds. For example, Pinelo et al. [50] reported that maceration at high temperatures increased the extraction of anthocyanins from the grape skin, as this practice enhanced both the solubility of the solute and the diffusion coefficient. Therefore, the differences in EC_50_ values of the grape seeds, pomace, and skins obtained during the winemaking processes and vinification lees may vary according to vinification techniques.

Isolated phenolics and/or extracts obtained from wine wastes (grape seeds, skins, and pomace) exhibited inhibitory effects against Gram-positive bacteria, Gram-negative bacteria, and fungi, including *B. cereus*, *S. aureus*, *Escherichia coli*, *S. typhimurium*, *Y. enterocolitica*, *L. monocytogenes*, *H. pylori*, and *C. jejuni* [51,52,53,54,55]. However, the TPC was not found to correlate with antimicrobial activities which suggested that the antimicrobial activity of wine lees may be related to the phenolic composition rather than the quantity of the phenolics [31,54,55]. It was suggested that anthocyanins which exhibited low MIC values (indicating high antimicrobial activities) have a lower antioxidant activity compared with other phenolics such as gallic acids [31]. Quercetin-methyl-glucoside (QMG) was found to correlate with the antimicrobial activity (*S. aureus*) (*p* < 0.05), which was consistent with the observation that QMG showed the highest concentration in the RL5 lees sample which exhibited the strongest antimicrobial activity against *S. aureus* (Table 2 and Table 5).

## 5. Conclusions

Various vinification techniques modified the extraction of phenolic compounds. More phenolic extraction to wine means correspondingly less phenolic compounds remaining in the grape marc. This is not true for wine lees. The lees samples in this study were obtained ranging from 1 to 5 months post alcoholic fermentation when the extraction processes were completed. It would be expected that vinification techniques that lead to more extraction into wine or juice would potentially result in (proportionally) more phenolics and other compounds bounded with dead yeast or yeast residues and would become part of wine lees. This highlights the fact that lees obtained from wines with a high phenolic content can be used as a potential source of phenolic compounds.

Knowledge of the characteristics of grape varieties and wine vinification techniques can provide some information on the potential composition and content in wine lees. In this study, the grape variety, harvest method, winemaking stages when the lees sample were obtained, additives, and vessels all seemed to play a part in determining the phenolic compositions of the lees. Further research is required to identify and quantify the influence of each individual vinification factor.

The results also showed a good linear relationship between the antioxidant activities and the TPC and TTC, indicating that lees can be a useful source of extracts with appreciable antioxidant activities. The PN lees had significantly higher values in the TPC, TTC, and higher antioxidant activities compared with the RL lees samples (Figure 1 and Figure 2 and Table 3), suggesting that red wine lees are an excellent source of phenolic compounds and antioxidant compared to white wine lees. The antimicrobial activity, on the other hand, is more likely to be dependent on the composition of the phenolic profile rather than the absolute quantity of the total phenolic content.

## Figures and Tables

**Table 1:** Information summary of the studied lees materials.

Sample ID	Variety	Vineyard			Fermentation			Postfermentation	
			Yeast Species	T (°C)	Maceration			Vessel	MLF (Y/N)
					Pre or Post	T (°C)	Vinification		
PN1	Pinot Noir	Pegasus Bay	Natural	34	Pre or Post	11–14	PO, P	Barrel	Y
PN2	Pinot Noir	Pegasus Bay	Natural	34	Pre or Post	11–14	PO, P	Barrel	Y
PN3	Pinot Noir	Pegasus Bay	Natural	34	Pre or Post	11–13	P	Barrel	Y
PN4	Pinot Noir	Pegasus Bay	Natural	33	Pre or Post	11–13	PO, P	Barrel	Y
PN5	Pinot Noir	Pegasus Bay	Natural	35	Pre or Post	10–13	PO, P	Barrel	Y
PN6	Pinot Noir	Pegasus Bay	Natural	34	Pre or Post	8–13	P	Barrel	Y
PN7	Pinot Noir	Felton Road	-	27–30	Pre or Post	15	-	Stainless steel tank	N
PN8	Pinot Noir	VinPro	RC212	30–32	Pre	8	PD	Barrel	Y
PN9	Pinot Noir	Omihi Hill	-	-	Pre or Post	10	-	Stainless steel tank	N
PN10	Pinot Noir	Omihi Hill	-	-	Pre or Post	10	-	Stainless steel tank	N
RL1	Riesling	Pegasus Bay	Vin13	16	Pre	-	-	Stainless steel tank	N
RL2	Riesling	Pegasus Bay	Vin13	17	-	-	-	Stainless steel tank	N
RL3	Riesling	Pegasus Bay	Vin13	16	-	-	-	Stainless steel tank	N
RL4	Riesling	VinPro	SIHL7	15	-	-	-	Stainless steel tank	N
RL5	Riesling	Omihi Hill	-	-	-	-	-	Stainless steel tank	N
RL6	Riesling	Omihi Hill	-	-	-	-	-	Stainless steel tank	N

“-“: information not available. Pre represents pre-fermentation maceration; post represents post-fermentation maceration; PO represents pump over; P represents plunging; PD represents punch down; and MLF (Y/N) represents with (Y) or without (N) malolactic fermentation.

**Table 2:** Phenolic profile of PN and RL lees extracts (mg/g extracts) quantified by LC-MS analysis.

Phenolic Compounds	Wine Lees (mg/g Extracts)															
	PN1	PN2	PN3	PN4	PN5	PN6	PN7	PN8	PN9	PN10	RL1	RL2	RL3	RL4	RL5	RL6
**Flavonoids**																
**Flavan-3-ols**																
Catechin	1.1 ± 0.0 ^c^	0.9 ± 0.0 ^bc^	1.7 ± 0.1 ^abc^	0.7 ± 0.0 ^c^	1.4 ± 0.1 ^bc^	1.7 ± 0.4 ^abc^	1.1 ± 0.1 ^bc^	0.2 ± 0.0 ^c^	5.9 ± 0.1 ^a^	2.5 ± 0.2 ^abc^	0.3 ± 0.2 ^c^	0.4 ± 0.0 ^c^	0.3 ± 0.0 ^c^	3.5 ± 0.3 ^ab^	1.7 ± 0.3 ^abc^	0.1 ± 0.0 ^c^
Epicatechin	0.8 ± 0.1 ^c^	0.6 ± 0.0 ^bc^	1.2 ± 0.0 ^abc^	0.5 ± 0.0 ^bc^	0.9 ± 0.1 ^abc^	0.9 ± 0.1 ^abc^	0.8 ± 0.0 ^abc^	0.5 ± 0.0 ^bc^	2.9 ± 0.0 ^a^	1.4 ± 0.0 ^ab^	-	0.1 ± 0.0 ^bc^	-	0.6 ± 0.1 ^bc^	0.5 ± 0.2 ^bc^	0.1 ± 0.0 ^bc^
Epicatechin gallate	0.4 ± 0.0 ^d^	0.2 ± 0.1 ^d^	0.4 ± 0.0 ^cd^	0.2 ± 0.1 ^d^	0.3 ± 0.1 ^cd^	0.5 ± 0.0 ^bcd^	0.4 ± 0.0 ^cd^	0.3 ± 0.0 ^cd^	0.7 ± 0.3 ^bcd^	0.5 ± 0.0 ^bcd^	0.1 ± 0.0 ^d^	0.1 ± 0.0 ^d^	-	1.3 ± 0.0 ^b^	3.6 ± 0.4 ^a^	1.7 ± 0.1 ^bc^
Procyanidin A	0.5 ± 0.1 ^cd^	0.5 ± 0.0 ^bcd^	0.7 ± 0.0 ^bcd^	0.3 ± 0.0 ^cd^	0.5 ± 0.1 ^bcd^	0.4 ± 0.0 ^cd^	0.4 ± 0.0 ^bcd^	0.2 ± 0.0 ^cd^	1.7 ± 0.1 ^b^	0.9 ± 0.0 ^bc^	0.1 ± 0.0 ^cd^	0.3 ± 0.1 ^bcd^	0.2 ± 0.0 ^cd^	-	5.1 ± 0.6 ^a^	0.4 ± 0.0 ^bcd^
Procyanidin B	0.8 ± 0.1 ^c^	0.6 ± 0.0 ^bc^	1.2 ± 0.0 ^ab^	0.6 ± 0.0 ^bc^	0.8 ± 0.0 ^bc^	1.0 ± 0.1 ^bc^	0.9 ± 0.0 ^abc^	0.5 ± 0.0 ^bc^	2.4 ± 0.0 ^a^	1.2 ± 0.1 ^ab^	0.2 ± 0.0 ^c^	-	-	0.3 ± 0.1 ^bc^	0.4 ± 0.2 ^bc^	-
Procyanidin C	-	-	0.1 ± 0.0 ^b^	-	-	-	-	-	-	-	-	1.2 ± 0.0 ^c^	1.2 ± 0.1 ^c^	3.2 ± 0.1 ^a^	3.7 ± 0.1 ^a^	1.8 ± 0.3 ^c^
**Flavonols**																
Quercetin	1.5 ± 0.1 ^d^	1.1 ± 0.1 ^de^	2.4 ± 0.1 ^cd^	2.1 ± 0.1 ^cde^	2.2 ± 0.1 ^cde^	3.0 ± 0.0 ^c^	0.6 ± 0.0 ^e^	0.7 ± 0.0 ^e^	4.4 ± 0.2 ^c^	1.0 ± 0.0 ^de^	6.8 ± 0.8 ^a^	0.7 ± 0.0 ^e^	1.5 ± 0.2 ^cde^	5.0 ± 0.1 ^b^	5.2 ± 0.7 ^ab^	0.8 ± 0.3 ^de^
Quercetin methyl-glucoside	0.6 ± 0.1 ^de^	0.5 ± 0.1 ^de^	0.5 ± 0.1 ^cde^	0.4 ± 0.0 ^de^	0.4 ± 0.0 ^de^	0.6 ± 0.1 ^cde^	0.5 ± 0.0 ^de^	0.5 ± 0.0 ^de^	0.9 ± 0.2 ^b^	0.6 ± 0.0 ^cd^	0.6 ± 0.0 ^cd^	0.2 ± 0.0 ^e^	0.6 ± 0.1 ^cd^	0.8 ± 0.0 ^bc^	4.2 ± 0.1 ^a^	1.0 ± 0.0 ^b^
**Nonflavonoids**																
**Hydroxybenzoic acids**																
Gallic acid	1.8 ± 0.0 ^ab^	1.9 ± 0.1 ^ab^	2.2 ± 0.0 ^a^	1.6 ± 0.1 ^b^	2.0 ± 0.0 ^ab^	1.8 ± 0.0 ^ab^	0.9 ± 0.0 ^c^	0.5 ± 0.0 ^cd^	1.7 ± 0.8 ^ab^	1.6 ± 0.0 ^b^	0.8 ± 0.0 ^c^	-	0.1 ± 0.1 ^d^	-	0.2 ± 0.0 ^cd^	-
**Hydroxycinnamic acids**																
Caftaric acid	1.2 ± 0.2 ^de^	1.1 ± 0.3 ^de^	1.4 ± 0.1 ^de^	1.7 ± 0.5 ^d^	1.3 ± 0.3 ^de^	1.8 ± 0.6 ^cd^	0.9 ± 0.2 ^de^	2.0 ± 0.1 ^bcd^	3.3 ± 1.4 ^b^	2.2 ± 0.1 ^bcd^	0.8 ± 0.2 ^de^	-	-	0.3 ± 0.1 ^e^	8.8 ± 0.6 ^a^	3.2 ± 0.1 ^bc^
Cinnamic acid	-	-	-	-	-	-	-	-	-	-	1.7 ± 0.2 ^a^	-	0.2 ± 0.0 ^b^	-	-	-
p-coumaric acid	-	-	-	-	-	-	-	-	-	-	2.4 ± 0.3 ^a^	0.1 ± 0.0 ^b^	0.3 ± 0.1 ^b^	0.4 ± 0.0 ^b^	0.1 ± 0.1 ^c^	-
**Stilbenes**																
Resveratrol	0.1 ± 0.0 ^b^	-	-	-	0.1 ± 0.0 ^b^	0.1 ± 0.0 ^b^	-	0.2 ± 0.0 ^b^	0.4 ± 0.2 ^a^	0.2 ± 0.0 ^b^	0.5 ± 0.1 ^a^	-	-	-	-	-

“-”: below detection limit. Means that do not share the same letter (a–e) are significantly different (*p* < 0.05).

**Table 3:** Mean antioxidant activity of wine lees samples was measured by DPPH radical scavenging activity (EC_50_) and oxygen radical absorbance capacity (ORAC).

Sample	EC_50_ (g FD Lees/g DPPH)	ORAC (mg TE/g FD Lees)
PN 1	9.9 ± 1.7 ^a^	475.0 ± 18.7 ^c^
PN 2	10.0 ± 0.3 ^a^	516.7 ± 21.0 ^cde^
PN 3	15.2 ± 0.7 ^a^	529.1 ± 62.1 ^cde^
PN 4	16.7 ± 0.2 ^a^	451.3 ± 80.7 ^bc^
PN 5	15.9 ± 0.7 ^a^	497.7 ± 60.8 ^cd^
PN 6	15.6 ± 0.1 ^a^	477.8 ± 18.3 ^cd^
PN 7	17.3 ± 0.1 ^a^	456.5 ± 7.9 ^bc^
PN 8	17.2 ± 1.7 ^a^	364.0 ± 29.4 ^b^
PN 9	11.5 ± 0.5 ^a^	602.4 ± 27.0 ^e^
PN 10	16.4 ± 0.7 ^a^	578.8 ± 24.0 ^de^
RL 1	107.3 ± 8.3 ^b^	104.5 ± 20.9 ^a^
RL 2	169.8 ± 0.5 ^de^	68.3 ± 5.1 ^a^
RL 3	251.9 ± 13.2 ^f^	59.8 ± 7.5 ^a^
RL 4	141.3 ± 2.5 ^c^	77.9 ± 2.2 ^a^
RL 5	179.7 ± 3.0 ^e^	70.1 ± 9.5 ^a^
RL 6	158.9 ± 4.3 ^d^	46.5 ± 2.3 ^a^

EC_50_: effective concentration, or the amount of sample required to decrease the initial concentration of DPPH• by 50%. TE: Trolox equivalent. Samples which do not share the same letter (a–f) are significantly different (*p* < 0.05).

**Table 4:** Pearson coefficient correlations between TPC, TTC, and antioxidant activity (DPPH and ORAC) of extracts obtained from different wine lees using 50% acidified solvent of ethanol (1% HCl).

Correlation	TPC	TTC	DPPH	ORAC
**TTC**	0.961			
**DPPH**	−0.904	−0.897		
**ORAC**	0.986	0.968	−0.914	

**Table 5:** Minimum inhibitory concentration (MIC) and minimum bactericidal concentration (MBC) values of PN and RL wine lees.

Extracts	Antibacterial/Antifungal Activity (MIC_50_) mg/mL					
	*S. aureus* ^1^		*E. coli* ^1^		*C. albicans* ^2^	
	(MIC) mg/mL	(MBC) mg/mL	(MIC) mg/mL	(MBC) mg/mL	(MIC) mg/mL	(MBC) mg/mL
PN1	1.56	3.13	-	-	-	-
PN2	3.13	6.25	-	-	-	-
PN3	3.13	6.25	25.00	-	-	-
PN4	3.13	6.25	-	-	-	-
PN5	6.25	6.25	-	-	-	-
PN6	1.56	3.13	12.50	-	-	-
PN7	3.13	6.25	-	-	-	-
PN8	3.13	3.13	12.50	12.50	-	-
PN9	3.13	6.25	-	-	-	-
PN10	3.13	6.25	-	-	-	-
RL1	1.56	3.13	6.25	6.25	**12.50**	12.50 *
RL2	6.25	12.5	25.0	25.0	50.00	50.00 *
RL3	1.6	3.13	6.25	6.25	-	-
RL4	3.13	6.25	6.25	6.25	**12.50**	**12.50**
RL5	**0.78**	**1.56**	**3.13**	**3.13**	-	-
RL6	3.13	6.25	12.5	12.5	-	-
	**Antibacterial/Antifungal Activity (MIC) µg/mL**					
**Antimicrobial agent**	*E. coli* ^1^		*S. aureus* ^1^		*C. albicans* ^2^	
Ampicillin	**3.125**		**0.781**			
Amphotericin B					**6.25**	

^1^ MIC values after 24 h of incubation with bacteria. ^2^ MIC values after 48 h of incubation with fungi. Bold font indicates the highest antibacterial and antifungal activity (MIC_50_) in each column. * different resistance abilities.

## Data Availability

Data are contained within the article.
